# Astragaloside IV suppresses histamine-induced inflammatory factors and mucin 5 subtype AC overproduction in nasal epithelial cells via regulation of inflammation-related genes

**DOI:** 10.1080/21655979.2021.1965813

**Published:** 2021-09-05

**Authors:** Jie Guo, Shuai Xu

**Affiliations:** Department of Otolaryngology-Head and Neck Surgery, Affiliated Luoyang Central Hospital of Zhengzhou University, Luoyang Henan, China

**Keywords:** Antihistamine, nasal mucosa, allergic rhinitis, muc5ac, inflammatory cytokine, rna-seq

## Abstract

Allergic rhinitis (AR) is a symptomatic allergic disease that leads to severe inflammation. Astragaloside IV (AS-IV) is a primary active component of *Astragalus membranaceus* and exerts immune-regulation and anti-inflammatory effects. However, the pharmacological effect of AS-IV in the nasal epithelial cells (NECs) has not been reported. The present study aimed to assess the effect of AS-IV on inflammatory cytokines and mucin 5 subtype AC (MUC5AC) overproduction in histamine (His)-stimulated NECs and its underlying mechanism. NECs were stimulated with or without His for 24 h in the absence or presence of AS-IV. The levels of inflammatory cytokines including IL-6, IL-8, MCP-1, IL-1β, granulocyte-macrophage colony-stimulating factor (GM-CSF), eotaxin, and MUC5AC were assayed. Our findings indicated that AS-IV inhibited His-evoked release and expression of inflammatory cytokines and MUC5AC in NECs. RNA-seq analyses indicated the significant changes in expression levels involved in inflammation genes upon treatment of His-induced NECs with AS-IV. Our findings indicated that AS-IV inhibited His-evoked inflammatory cytokines secretion and MUC5AC overproduction in NECs, which were partly mediated by regulation of inflammation-related genes. Therefore, our findings provided a scientific basis for the development of AS-IV as an effective agent for clinical therapeutic strategy in the treatment of AR.

## Introduction

Allergic rhinitis (AR) is a common chronic allergic respiratory disease among children and adults, which occurs in nasal mucosa by the overreaction of allergens [1,2]. According to epidemiological research, the prevalence of AR, which affects 500 million people worldwide, is increasing at an alarming rate [3]. Although AR is not fatal, patients with AR continuously suffer from symptoms, including nasal overﬂow, rhinorrhea, respiratory obstruction, nasal itching, sneezing, and nasal congestion [4]. Besides, AR patients may also be affected by mood disorders, sleep disorders, and deterioration in social relationships [5]. The symptoms of AR are induced by the secretion of histamine (His), pro-inflammatory cytokines, and allergic, which could induce secretion of tears, and evoke vascular dilation [6]. The airway epithelium is the first line of host defense. Increasing evidence indicated that nasal epithelial cells (NECs) play a vital role in the etiopathogenesis of AR [7]. It has been demonstrated that chronic inflammation of NECs is involved in the pathogenesis of AR [8]. Pro-inflammatory cytokines including interleukin (IL)-6 and IL-8 are highly upregulated in NECs after the stimulation of *Dermatophagoides pteronyssinus* 1 [7]. Moreover, the inhibition of inflammatory cytokines secretion contributes to suppressing allergic responses in AR [9]. Mucins are highly glycosylated macromolecules secreted by goblet cells in epithelial tissues. However, the overproduction of mucins is a pathognomonic characteristic in chronic airway diseases, which results in the dysfunction of mucociliary in the airways [10]. Mucin 5 Subtype AC (MUC5AC) is considered to be the important mucin in the airway, and the secretion and expression of MUC5AC are increased in nasal allergic inflammation [11]. Therefore, targeting the inflammatory cytokines and MUC5AC might be a potential therapeutic strategy in the treatment of AR. Currently, there are lots of agents that are used for the treatment of AR, including immunotherapy, leukotriene receptor antagonists, antihistamines, and nasal steroids. However, those drugs would cause a frequency of side effects, and most patients stop using those agents. Therefore, innovative, safe, and effective therapeutic strategies are still urgently needed for AR patients. In recent years, more and more research has focused on the development of natural products derived from medicinal plants for the management of the allergic respiratory disease [12–14].

*Astragalus membranaceus* is an herb, which has long been used in China for the treatment of numerous diseases, including kidney diseases, skin diseases, hepatitis, allergic rhinitis, and cardiovascular diseases [15,16]. Astragaloside IV (As-IV) is the primary bioactive constituent derived from the *Astragalus membranaceus* [17]. Accumulated researches indicated that As-IV has diverse pharmacologic activities, such as anti-inflammatory, immunomodulatory, antiviral, and antiapoptosis effects [18]. It has been reported that As-IV could alleviate airway inflammation and airway hyperresponsiveness in an ovalbumin-evoked asthma model [19]. Besides, As-IV improved pulmonary function via suppressing the secretion of IL-6 and tumor necrosis factor-alpha (TNF-α) in the chronic hypoxia-induced pulmonary hypertension model [20]. However, to the best of our knowledge, the effects of AS-IV on inflammatory cytokines, MUC5AC secretion, and gene expression in NECs have not been reported.

Based on these reports, we speculated AS-IV may exert a therapeutic effect in AR disease. Therefore, a NECs cells model of AR was established to investigate the effect of AS-IV on His-evoked inflammatory response and MUC5AC overproduction. Besides, the RNA-seq and qRT-PCR experiments were used to further explore the potential targeted genes and pathways of AS-IV against AR.

## Materials and methods

### NECs cultures

NECs were purchased from Jennio Biotech Co., Ltd. (Guangzhou, China) and maintained in a humidified chamber with 5% CO_2_/95% air at 37 °C. NECs were incubated in bronchial epithelial cell growth medium (BEGM) medium (Lonza, Walkersville, Md., USA) contained with 100 U/ml penicillin (Sigma-Aldrich, MO, USA), 100 μg/ml streptomycin (Sigma-Aldrich, MO, USA), and 10% fetal bovine serum (Sigma-Aldrich, MO, USA).

### Cell viability measurement

3-[4,5-dimethylthiazol-2-yl]-2,5 diphenyl tetrazolium bromide (MTT) method was performed to evaluate the cell viability based on a previous study [21]. Briefly, NECs were seeded into 96-well plates (5 × 10^3^ cells/well). After 24 h of pre-incubation, NECs were cultured with different concentrations of His (0, 0.025, 0.05, and 0.1 µM; Sigma-Aldrich, MO, USA) or AS-IV (0, 20, 40, 60, and 80 µM; > 98% purity; Aladdin, Shanghai, China). After 24 and 48 h of incubation, 20 µL of MTT solution (Sigma-Aldrich, MO, USA) was transferred to each well and cultured at 37 °C for another 4 h. Then, the medium was discarded and the generated formazan crystal was dissolved in 200 µL of dimethyl sulfoxide (Sigma-Aldrich, MO, USA). Finally, the absorbance was measured in a microplate reader (Bio-Rad, Hercules, CA, USA) at 570 nm.

### Treatment of NECs with AS-IV and His stimulation

A NECs cells model of AR was established by His stimulation based on a previous study [22]. The NECs were pretreated with AS-IV (0, 20, 40, and 60 µM) or BAY 11–7083 (3 µM) for 30 min. BAY 11–7082, a nuclear factor-kappaB (NF-κB) inhibitor (NF-κBi), was obtained from Beyotime (Shanghai, China). Subsequently, NECs were either unstimulated or stimulated with His (0.1 µM) for 24 h in a BEGM medium. His and AS-IV were dissolved in 0.1% dimethyl sulfoxide before the experiment. NECs were dissolved in the same amount of 0.1% dimethyl sulfoxide in the control group. After 24 h of incubation, cell pellets were collected for further analysis.

### RNA sequencing (RNA-seq) analysis

The NECs were seeded in cell plates for 24 h. The NECs were pretreated with AS-IV (60 µM) for 30 min. Subsequently, NECs were either unstimulated or stimulated with His (0.1 µM) for 24 h in a BEGM medium. Total RNA was extracted from NECs using Trizol reagent (Invitrogen, CA, USA). The PCR amplification and sequencing were performed on the GPL16791 Illumina HiSeq 2500 (Homo sapiens). Fold change ≥ 2 and *p* < 0.05 were used as the criteria for the differentially expressed genes (DEGs) and enriched via KEGG analysis.

### Measurement of inflammatory cytokines releasing from NECs cells

NECs cell supernatant was collected by centrifugation at 4,000 *g* for 5 min at 4 °C after 24 h of co-treatment for inflammatory cytokines measurement. The levels of IL-6, IL-8, monocyte chemoattractant protein-1 (MCP-1), interleukin-1beta (IL-1β), granulocyte-macrophage colony-stimulating factor (GM-CSF), and eotaxin in cell culture supernatant were detected using commercially available enzyme-linked immunosorbent assay (ELISA) kits (R&D Systems, MN, USA) and conducted according to the manufacturer’s protocols.

### Assay of MUC5AC mucin

NECs cell supernatant was obtained by centrifugation at 4,000 *g* for 5 min at 4 °C after 24 h of co-treatment for MUC5AC assay. MUC5AC protein released from cell culture supernatant was detected using Human MUC5AC ELISA kits (NeoMarkers, CA, USA) and performed according to the manufacturer’s protocols.

### RNA extraction and real-time quantitative reverse transcription-PCR (qRT-PCR)

Total RNA from NECs was extracted using Trizol reagent (Invitrogen, CA, USA) and purified by the RNeasy kit (Qiagen Inc, CA, USA) according to the manufacturer’s instructions. Then, reverse-transcription of total RNA was performed by SuperScript III Reverse Transcriptase (Invitrogen, CA, USA). Then, the qRT-PCR was implemented on the ABI Prism 7300 Detection System (Applied Biosystems, CA, USA) by the SYBR Green qPCR Super Mix-UDG kit (Invitrogen, CA, USA). Glyceraldehyde-3-phosphate dehydrogenase (GAPDH) was used for internal calibration. The comparative 2^−ΔΔCt^ method was used to calculate the relative mRNA expression. Primer sequences were displayed in Table 1.

**Table 1.** Primer sequences for quantitative real-time RNA

Genes	Forward primer	Reverse primer
CXCL11	5ʹ-ATGAGTGTGAAGGGCATGGC-3’	5ʹ-TCACTGCTTTTACCCCAGGG-3’
CXCL2	5ʹ-GCTGCTGCTCCTGCTTCTAGTG-3’	5ʹ-AGGTGAATTCCTTGCACGGTCTG-3’
CCL3	5ʹ-CATGGCGCTCTGGAACGAA-3’	5ʹ-TGCCGTCCATAGGAGAAGCA-3’
TNF	5ʹ-GCACTGAGAGCATGATCCGAGAC-3’	5ʹ-CGACCAGGAGGAAGGAGAAGAGG-3’
IL-1B	5ʹ-AAGTGATGGCTAACTACGGTGACAAC-3’	5ʹ-GCTTCTCCACTGCCACGATGAC-3’
IL-18	5ʹ-GCTTGAATCTAAATTATCAGTC-3’	5ʹ – GAAGATTCAAATTGCATCTTAT-3’
MUC5AC	5ʹ-CGACAACTACTTCTGCGGTGC-3’	5ʹ-GCACTCATCCTTCCTGTCGTT-3’
NF-κB	5ʹ-AGCACCAAGACCGAAGCAA-3’	5ʹ-TCTCCCGTA ACCGCGTAGTC-3’
GAPDH	5ʹ-CAACTTTGGCATTGTGGAAGG-3’	5ʹ-ACACATTGGGGGTAGGAACAC-3’

### Statistical Analysis

All results were shown as mean ± SD. Data analysis was carried out using statistical software (GraphPad Software, Inc., La Jolla, CA, USA). The statistical significance of differences among experimental groups was evaluated using a one-way analysis of variance (ANOVA) followed by Dunnett’s post hoc test. A value of P < 0.05 was considered statistical significance.

## Results

In the present study, we hypothesized that AS-IV could exert beneficial effects in NECs induced by His. A NECs cells model of AR was established to validate our hypothesis. Our findings indicated that AS-IV inhibited His-induced inflammatory cytokines secretion and MUC5AC overproduction by regulation of inflammation-related genes.

### NECs cell viability measurement

**Figure 1.**
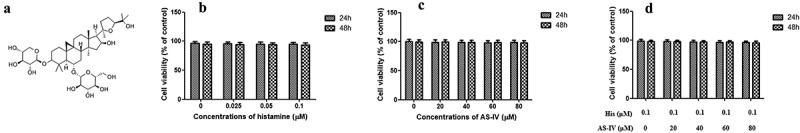
Effect of histamine, and AS-IV on cell viability of nasal epithelial cells. Chemical structure of AS-IV (a). Effect of histamine on cell viability of nasal epithelial cells (b). Effect of AS-IV on cell viability of nasal epithelial cells (c). Effect of His + AS-IV on cell viability of nasal epithelial cells (d). The results were expressed as the mean ± SD of three independent experiments

In our study, an MTT assay was carried out to exclude the possibility that the impact of His or AS-IV on the cell viability of NECs. The chemical structure of AS-IV was shown in Figure 1a. As shown in Figure 1b-d, after treatment with different concentrations of His (0–0.1 µM) for 24, and 48 h, or AS-IV (0–80 µM) for 24, and 48 h for 24, and 48 h, no significant differences were observed in NECs cell viability. Our findings indicated that the appointed concentrations of His, and AS-IV were nontoxic effects on the cell viability of NECs.

### AS-IV inhibited His-evoked inflammatory cytokine production in NECs

**Figure 2.**
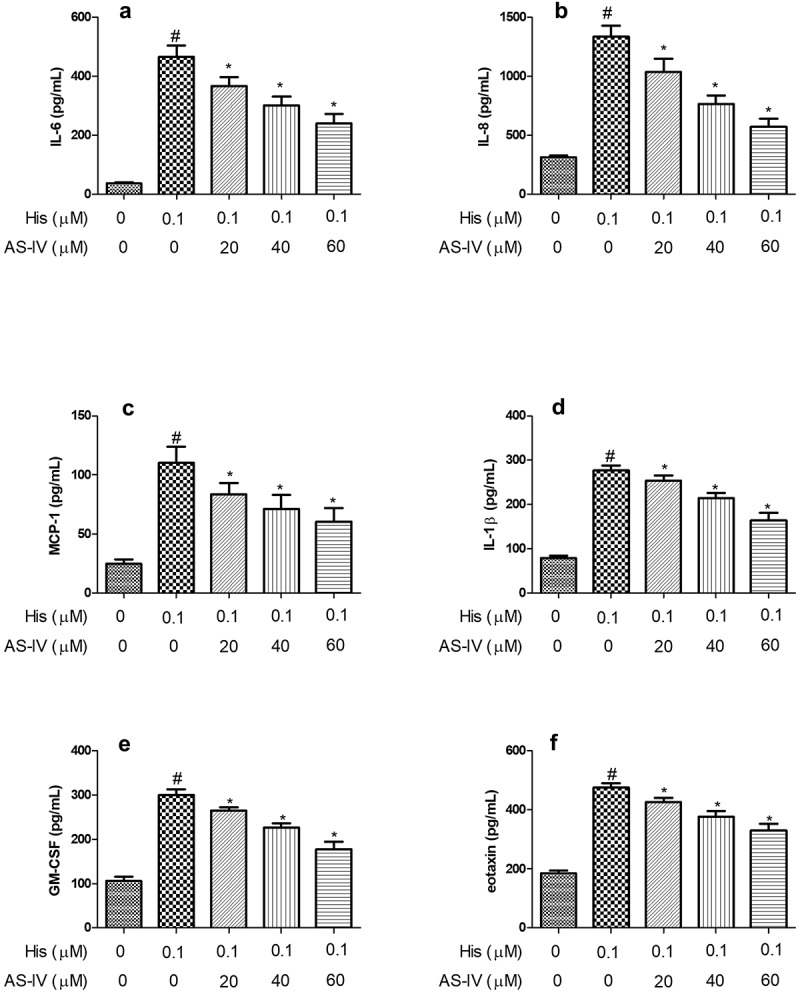
AS-IV inhibited His-evoked inflammatory cytokine production in nasal epithelial cells. The levels of IL-6 (a), IL-8 (b), MCP-1 (c), IL-1β (d), GM-CSF (e), and eotaxin (f) were assayed by ELISA. The results were expressed as the mean ± SD of three independent experiments. ^#^*P* < 0.01 vs. control group, *P < 0.01 vs. His group

Previous reports have shown that pro-inflammatory cytokines, including IL-6, IL-8, MCP-1, and IL-1β are up-regulated and play a vital role in AR [23,24]. Therefore, the pro-inflammatory cytokines were measured in NECs to investigate the effect of AS-IV on the inflammatory response. As shown in Figure 2a-d, the secretion of IL-6, IL-8, MCP-1, and IL-1β in NECs supernatant was increased after His induction, and pre-treatment with AS-IV (20, 40, and 60 µM) inhibited the production of IL-6, IL-8, MCP-1, and IL-1β evoked by His stimulation, indicating that AS-IV suppressed His-evoked inflammation response in NECs.

### AS-IV suppressed His-evoked GM-CSF and eotaxin secretion in NECs

As shown in Figure 2e-f, the secretion of GM-CSF and eotaxin in NECs supernatant were increased after His induction, and pre-treatment with AS-IV (20, 40, and 60 µM) inhibited the secretion of GM-CSF, and eotaxin evoked by His stimulation.

### AS-IV suppressed His-evoked MUC5AC secretion in NECs

**Figure 3.**
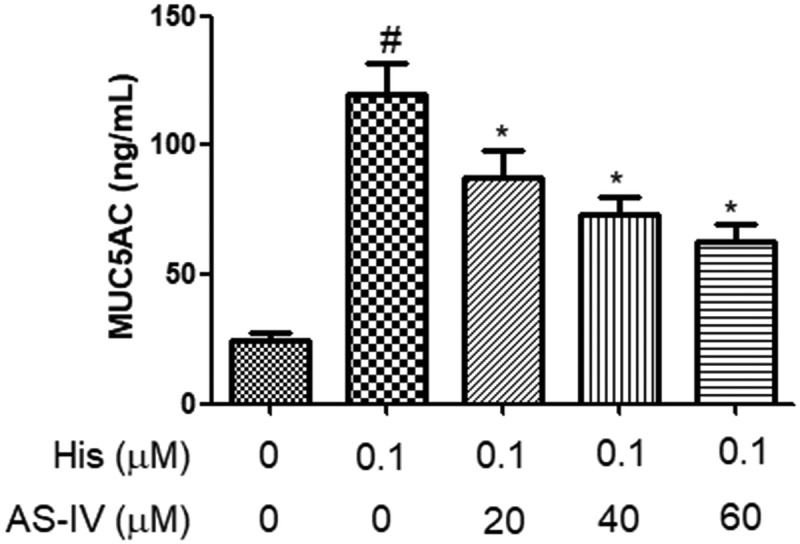
AS-IV suppressed His-evoked MUC5AC overproduction in nasal epithelial cells. The contents of MUC5AC were assayed using ELISA. The results were expressed as the mean ± SD of three independent experiments. ^#^*P* < 0.01 vs. control group, *P < 0.01 vs. His group

As shown in Figure 3, the secretion of MUC5AC in NECs supernatant was increased after His induction, and pre-treatment with AS-IV (20, 40, and 60 µM) inhibited the secretion of MUC5AC evoked by His stimulation, indicating that AS-IV suppressed His-evoked MUC5AC secretion in NECs.

### Differentially expressed genes (DEGs) profiling by RNA-Seq

**Figure 4.**
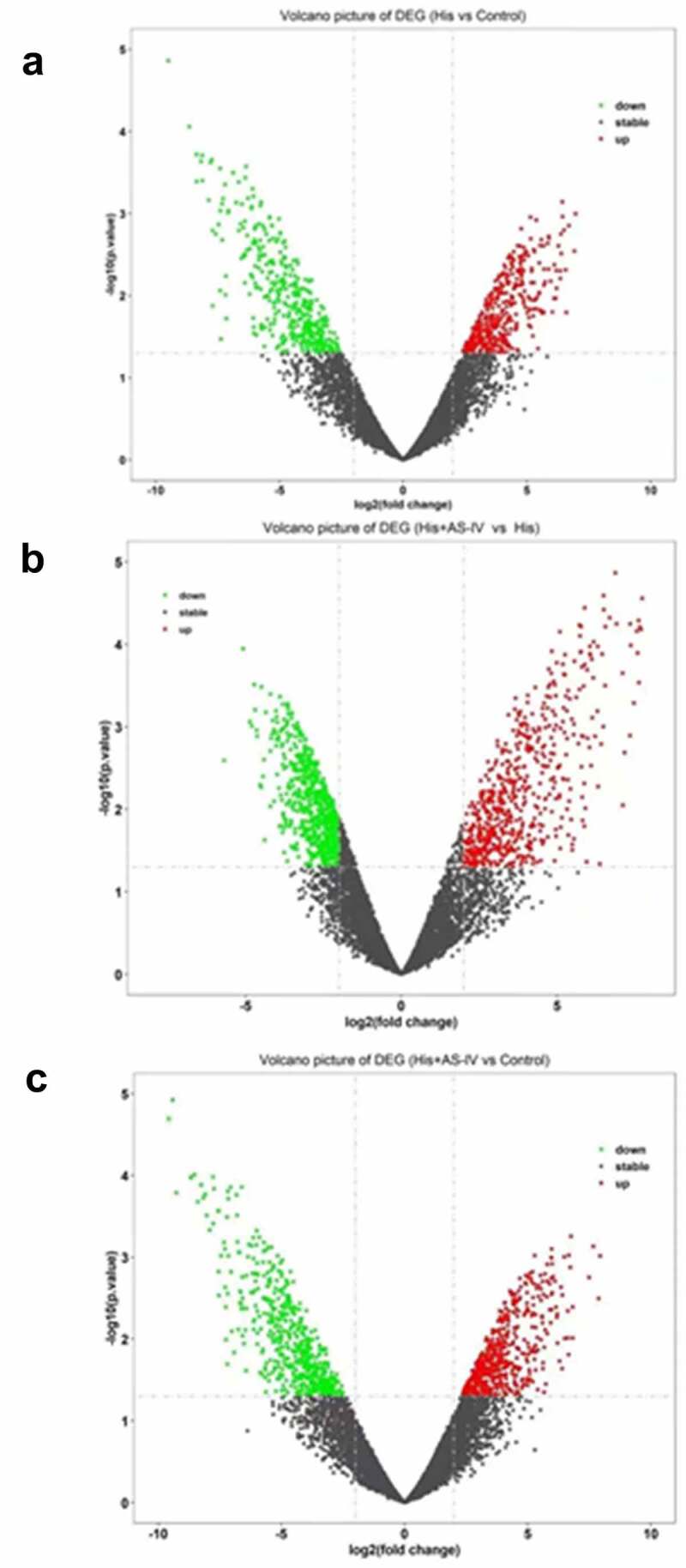
Volcano plot showing DEGs between two groups (A, B, and C). Green, gray, and red dots in the Volcano plot showed significantly downregulated genes, insignificantly DEGs, upregulated genes, respectively. The DEGs with fold change ≥ 2.0 and *p* < 0.05 and are shown

**Figure 5.**
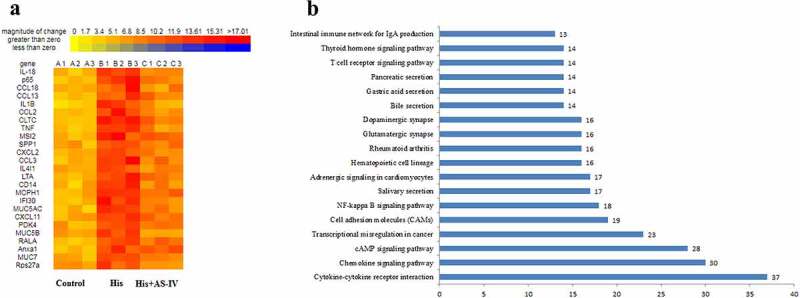
Heatmap diagram indicating that the common DEGs in the three groups (a). Red and yellow squares in the heatmap showed mRNAs with up-regulated and down-regulated expression levels, respectively. KEGG pathway analyses of the overlapping genes (b)

**Figure 6.**
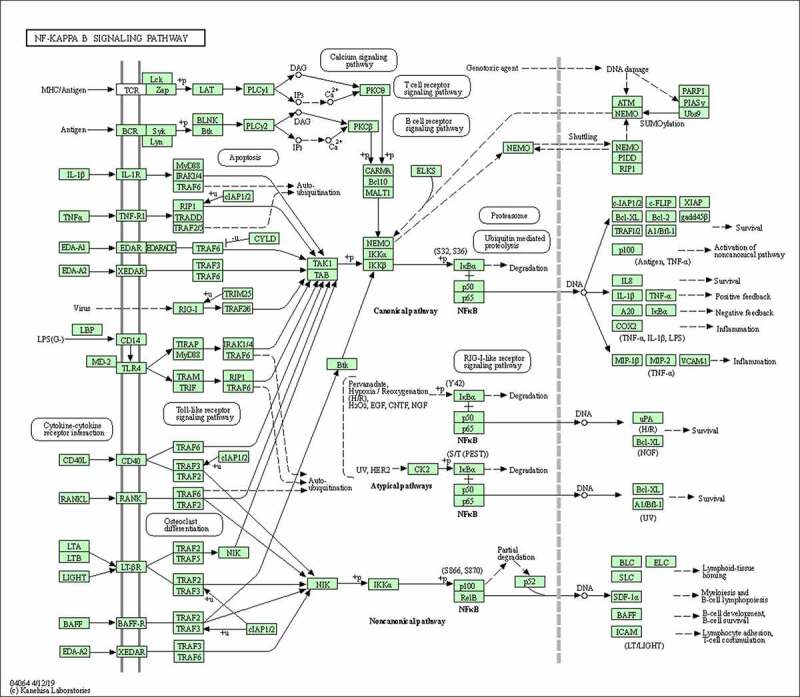
Enrichment pathway of KEGG in NF-KAPPA B signaling pathway

As shown in Figure 4, RNA-SEQ analysis was carried out using His-induced NECs treated with AS-IV. A total of 781 mRNAs with statistically significant differences were screened out in the His group relative to the control group (Figure 4a): there were 400 downregulated genes (green dots) and 381 upregulated genes (red dots). A total of 1215 mRNAs with statistically significant differences were screened out in the His group relative to the His+AS-IV group (Figure 4b): there were 636 downregulated genes (green dots) and 579 upregulated genes (red dots). A total of 1084 mRNAs with statistically significant differences were screened out in the control group relative to the His+AS-IV group (Figure 4c): there were 551 downregulated genes (green dots) and 533 upregulated genes (red dots). Besides, as shown in Figure 5a, the heat map indicating the top 25 DEGs, and most of these were responsible for inflammatory responses and inflammatory cytokines. The expression levels of inflammation-related genes, such as IL-18, p65, chemokine (C-C Motif) ligand 18 (CCL18), IL-1B, TNF, etc., were up-regulated by His and were down-regulated after AS-IV treatment. Moreover, Kyoto Encyclopedia of Genes and Genomes (KEGG) analysis (Figure 5b) indicated that genes related to inflammatory pathways, such as cytokine-cytokine receptor interaction, chemokine signaling pathway, and NF-kappa B signaling pathway (Figure 6). These results indicated that the overexpression of inflammation-related singling pathways evoked by His was down-regulated by AS-IV.

### AS-IV suppressed the His-induced overexpression of inflammation-related genes

**Figure 7.**
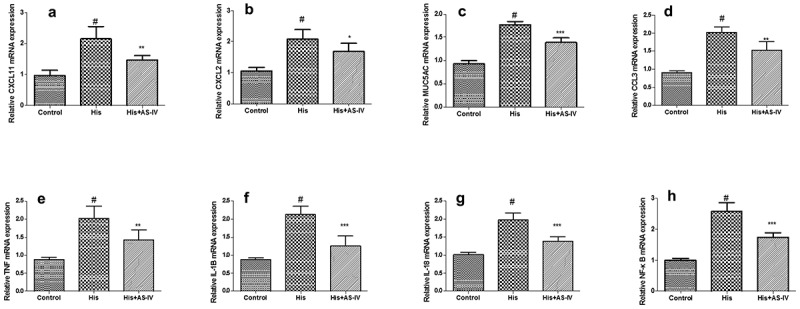
Validation of RNA-SEQ results using quantitative real-time polymerase chain reaction (qRT-PCR) analysis. The results were expressed as the mean ± SD of three independent experiments. ^#^*P* < 0.01 vs. control group, *P < 0.01 vs. His group

As shown in Figure 7, inflammation-related genes were randomly selected to verify the RNA-seq data by qRT-PCR. Our findings showed that the expression of C-X-C motif chemokine ligand 11 (CXCL11), C-X-C motif chemokine ligand 2 (CXCL2), MUC5AC, chemokine (C-C Motif) ligand 3 (CCL3), TNF, IL-1B, IL-18, and NF-κB were significantly increased in the His group, and down-regulated by AS-IV. These findings are consistent with the results of transcriptome sequencing, showing that the RNA-seq results are reliable, and further demonstrating that AS-IV can inhibit the inflammation-related gene overexpression induced by His.

***Suppression of NF-κB signaling pathway inhibited His-evoked secretion and expression of inflammatory cytokines in NECs***

**Figure 8.**
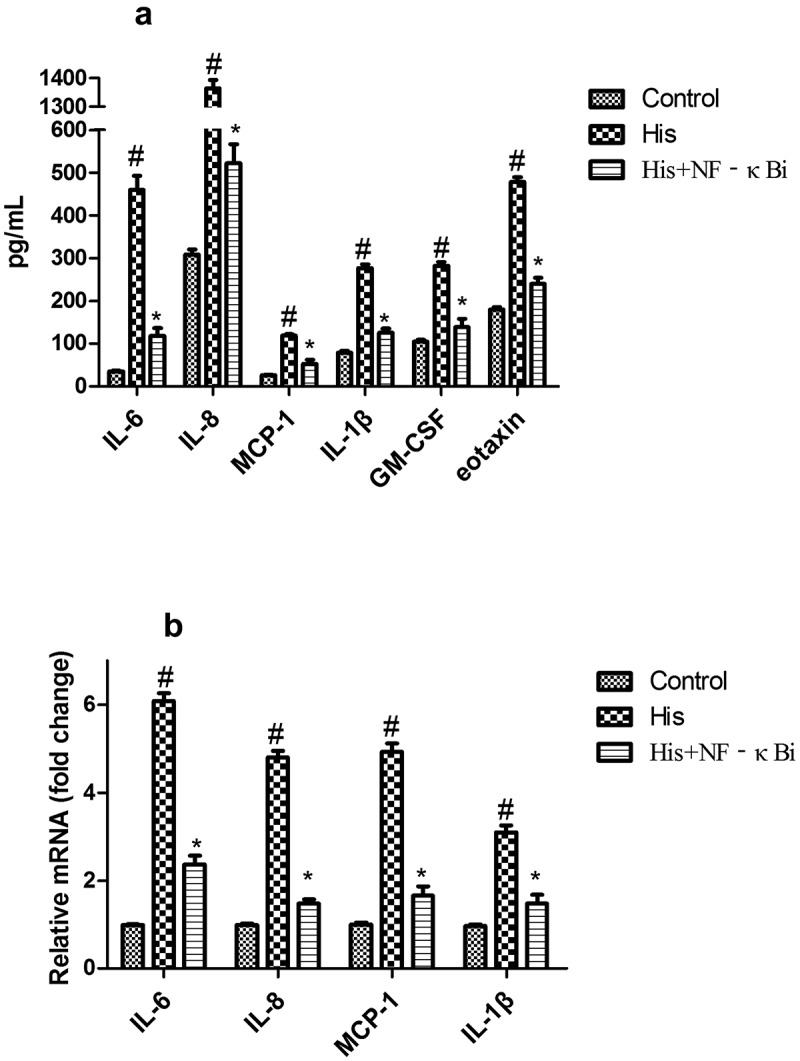
Suppression of the NF-κB signaling pathway decreased His-evoked inflammatory cytokine response in nasal epithelial cells. The effect of NF-κB inhibitor (NF-κBi) on His-evoked secretion of IL-6, IL-8, MCP-1, IL-1β, GM-CSF, and eotaxin (a). The effect of NF-κBi on His-evoked mRNA expression of IL-6, IL-8, MCP-1, and IL-1β (b). The results were expressed as the mean ± SD of three independent experiments. ^#^*P* < 0.01 vs. control group, *P < 0.01 vs. His group

Moreover, we also investigated the role of the NF-κB pathway in His-evoked inflammatory cytokines secretion and expression in NECs. As shown in Figure 8, our findings showed that NF-κBi treatment alleviated His-evoked secretion and expression of inflammatory cytokines in NECs, indicating that AS-IV alleviated His-evoked inflammatory response partly via suppression of NF-κB signaling pathway in NECs.

## Discussion

AR is a type of nasal inflammation induced by inhaled allergens [25]. His is an important inflammatory mediator in the pathogenesis of allergic inflammation, which has been reported to elicit an increase in the secretion of pro-inflammatory mediators and over-expressions of adhesion molecules [26]. Therefore, His is usually used for the establishment of the AR model *in vitro*. In our research, NECs were stimulated with His. Our findings demonstrated that His-evoked the production and expression of pro-inflammatory mediators (IL-6, IL-8, MCP-1, IL-1β, GM-CSF, and eotaxin), and MUC5AC, showing that an AR model was successfully established *in vitro*.

As-IV is the primary bioactive compound derived from the *Astragalus membranaceus*, has multiple pharmacological activities. It has been reported that AS-IV attenuates cognitive impairments partly through its anti-inflammatory activities via the suppression of inflammasome overactivation and the Toll-Like Receptor 4 (TLR4) signaling pathway [27]. And AS-IV protects against lipopolysaccharide-evoked injure by regulating miR-203/MyD88 [28]. In another research, AS-IV attenuates the symptoms of ovalbumin-induced allergic rhinitis via regulating the expression levels of GATA binding protein 3, forkhead box protein 3, and T cells [29]. However, no report had focused on the pharmacological effects of AS-IV on AR in His-induced NECs and its potential mechanism has not been clarified. In the present research, for the first time, our results indicated that AS-IV inhibited His-evoked secretion and expression of pro-inflammatory cytokines, and MUC5AC, implying that AS-IV attenuated inflammatory response and MUC5AC overexpression in NECs.

AR is characterized by more and more inflammatory factors in lesions, which result in itching, rhinorrhea, and sneezing [30]. It has been reported that activated mast cells generate chemokines, growth factors, cytokines, and mediators that result in the progression of AR [31]. Pro-inflammatory cytokines, such as IL-6, IL-8, MCP-1, and IL-1β, play an important role in allergen-caused AR and these mediators could be produced by inflammatory cells [31]. GM-CSF and eotaxin are known as pro-inflammatory factors in nasal inflammation that are generated and secreted by fibroblasts, infiltrating leukocytes, and airway epithelial cells in response to inflammatory mediators and allergens [32]. Previous reports have reported that the secretion of IL-6, IL-8, GM-CSF, and eotaxin were significantly increased *in vitro* AR model by stimulating NECs with inflammatory mediators [33,34]. In the present study, we found that His increased the levels of IL-6, IL-8, GM-CSF, and eotaxin in NECs. Moreover, AS-IV suppressed the secretion and expression of these mediators in His-stimulated NECs. It has been reported that MUC5AC is a primary member of mucoprotein and is up-regulated in airway epithelial cells due to the stimulation of the airway mucosa to pollutants, pathogens, and allergens [35,36]. Besides, MUC5AC secretion and expression were increased in His-stimulated NECs [34]. In the present study, we observed that His increased the secretion and expression of MUC5AC in NECs. Besides, AS-IV inhibited the secretion and expression of MUC5AC in His-stimulated NECs, indicating that AS-IV might be effective in the treatment of AR disease.

Activation of the NF-κB pathway could cause the up-regulation of inflammation-related genes [37]. Hence, inhibition of the NF-κB signaling pathway is an effective therapy for the treatment of inflammation-linked diseases such as AR. A previous report has indicated that Glycyrrhizin suppressed His evoked inflammatory response via inactivating the NF-κB pathway in NECs [22]. The suppression of the NF-κB pathway inhibited the secretion of IL-6, IL-8, MCP-1, IL-1β, GM-CSF, and eotaxin in the His-stimulated NECs. Therefore, we inferred that AS-IV might be considered as a potential candidate drug for the treatment of AR via inhibition of the NF-κB pathway *in vitro*.

Although the present study demonstrated that AS-IV exerted therapeutic effects against AR. However, there were several defects in our study. Firstly, our research was carried out in NECs, and animal AR models are needed to further verify these findings. Secondly, AS-IV could inhibit His-induced inflammation and MUC5AC overproduction in NECs, the detailed mechanism action of AS-IV remains more experiments to validate. Hence, it is necessary to address those defects in future experiments.

## Conclusion

In conclusion, our results revealed that AS-IV inhibited the secretion and expression of IL-6, IL-8, MCP-1, IL-1β, and MUC5AC in the His-stimulated NECs. Besides, AS-IV treatment inhibited His-evoked upregulation of inflammation-related genes. NF-κBi attenuated His-evoked secretion and expression of IL-6, IL-8, MCP-1, and IL-1β in the His-stimulated NECs. The findings implied that AS-IV suppressed His-evoked inflammation response and upregulation of MUC5AC via inhibition of inflammation-related genes *in vitro*. Hence, our results provided a scientific basis for the development of AS-IV as an effective drug for clinical therapeutic strategy in the treatment of AR.

## Data Availability

The datasets generated for this study are available on request to the corresponding author.

## Highlights

(1) AS-IV may be considered a novel drug for the treatment of AR.

(2) AS-IV inhibits His-induced inflammation in NECs.

(3) AS-IV inhibits MUC5AC overproduction of NECs induced by His.

(4) AS-IV regulates inflammation-related genes in His-stimulated NECs.
